# mTOR Overactivation and Compromised Autophagy in the Pathogenesis of Pulmonary Fibrosis

**DOI:** 10.1371/journal.pone.0138625

**Published:** 2015-09-18

**Authors:** Yao-Song Gui, Lianmei Wang, Xinlun Tian, Xue Li, Aiping Ma, Weixun Zhou, Ni Zeng, Ji Zhang, Baiqiang Cai, Hongbing Zhang, Jing-Yu Chen, Kai-Feng Xu

**Affiliations:** 1 Department of Respiratory Medicine, Peking Union Medical College Hospital, Peking Union Medical College and Chinese Academy of Medical Sciences, Beijing, China; 2 State Key Laboratory of Medical Molecular Biology, Department of Physiology and Pathophysiology, Institute of Basic Medical Sciences, Peking Union Medical College and Chinese Academy of Medical Sciences, Beijing, China; 3 Institute of Chinese Materia Medica, China Academy of Chinese Medical Sciences, Beijing, China; 4 Department of Pathology, Peking Union Medical College Hospital, Peking Union Medical College and Chinese Academy of Medical Sciences, Beijing, China; 5 Department of Thoracic Surgery, Wuxi People’s Hospital affiliated to Nanjing Medical University, Wuxi, China; University of Allabama at Birmingham, UNITED STATES

## Abstract

The mammalian target of rapamycin (mTOR) signaling pathway in pulmonary fibrosis was investigated in cell and animal models. mTOR overactivation in alveolar epithelial cells (AECs) was achieved in the conditional and inducible *Tsc1* knock-down mice SPC-*rtTA*/TetO-*Cre/Tsc1* ^*fx/+*^ (STT). Doxycycline caused *Tsc1* knock-down and consequently mTOR activation in AECs for the STT mice. Mice treated with bleomycin exhibited increased mortality and pulmonary fibrosis compared with control mice. In wild-type C57BL/6J mice, pretreatment with rapamycin attenuated the bleomycin-mediated mortality and fibrosis. Rapamycin-mediated mouse survival benefit was inhibited by chloroquine, an autophagy inhibitor. Autophagosomes were decreased in the lungs after bleomycin exposure. Rapamycin induced the production of autophagosomes and diminished p62. We concluded that mTOR overactivation in AECs and compromised autophagy in the lungs are involved in the pathogenesis of pulmonary fibrosis. The suppression of mTOR and enhancement of autophagy may be used for treatment of pulmonary fibrosis.

## Introduction

Idiopathic pulmonary fibrosis (IPF) is a chronic, progressive and fatal fibrotic lung disease with unknown causes and poor prognosis [1]. Although a great amount of research has been reported, only few therapies have proven to be effective [2, 3].

The PI3K/AKT/mammalian target of rapamycin (mTOR) signaling pathway is a core signaling pathway in cells that regulates cell growth, proliferation, motility, and survival [4]. mTOR, which belongs to the phosphatidylinositol 3-kinase-related kinase protein family, is a serine/threonine protein kinase that consists of two protein complexes, i.e., rapamycin-sensitive (mTOR complex 1, mTORC1) and rapamycin-insensitive protein complexes (mTORC2) [4, 5]. mTOR overactivation has been reported in human diseases, such as lymphangioleiomyomatosis, tuberous sclerosis complex, Alzheimer’s disease, obesity, diabetes and cancer [6–12].

Abnormal mTOR pathway activation is also involved in fibrotic diseases, such as cardiac fibrosis [13, 14], liver fibrosis [15], and kidney fibrosis [16, 17]. There is growing evidence to support the idea that the mTOR signaling pathway plays a key role in pulmonary fibrosis [18–21].

Injury and reprogramming in alveolar epithelial cells (AECs) are regarded as critical steps in the pathogenesis of IPF [22]. In this study, we used an *in vivo* model of inducible conditional *Tsc1* gene knock-down in AECs. mTOR overactivation in AECs has been found to be an important mechanism involved in bleomycin-mediated pulmonary injury and fibrosis. In wild-type C57BL/6J mice, pre-treatment with the mTOR inhibitor rapamycin attenuated bleomycin-mediated lung injury and improved survival. The benefit of rapamycin could be reversed by chloroquine, an autophagy inhibitor. Our study indicated that mTOR overactivation in AECs and autophagy dysfunction contributes to the pathogenesis of pulmonary fibrosis.

## Materials and Methods

### Human lung samples

Three IPF lung and two healthy human lung samples were used. Human tissues were donated by patients receiving lung transplantation and by unused healthy lung from donors for transplantation. IPF diagnoses were made according to American Thoracic Society guideline [1]. All subjects included in the study provided written informed consent. The study protocol was approved by the Ethics Committee of Peking Union Medical College Hospital and Wuxi People’s Hospital.

### Cell cultures and treatments

The human fetal lung fibroblast cell line MRC5 was purchased from the cell resource center of the Chinese Academy of Medical Sciences. Primary lung fibroblasts (PLF) were isolated from healthy human lungs. Briefly, fresh harvested lung tissue samples were maintained in sterile phosphate-buffered saline and cut into small pieces (1 mm^3^). Then, samples were placed in 25 cm^2^ flasks containing Dulbecco's Modified Eagle Medium (GIBCO) supplemented with 10% fetal bovine serum (Hyclone), penicillin (100 U/mL), and streptomycin (100 μg/mL). Lung tissue samples were cultured at 37°C in a humidified atmosphere of 5% CO_2_, and spindle-like fibroblasts started growing from tissue samples on day 2 to 3. The outgrowth of fibroblasts took approximately 2 weeks, and then cells were removed by aspiration, allowing cells to reach confluence. Lung fibroblasts were identified by morphology and immunofluorescence (data not shown). Fibroblasts between passages 5 and 8 were used in our experiments. Confluent cultures of cells (including MRC5 and PLF) were maintained in serum-free DMEM containing 0.1% FBS for 24 hours. The cells were then incubated with TGF-β1 (R&D) at a concentration of 5 ng/ml for 24 hours. Afterward, the cells were harvested for Western blot analysis.

### Mouse strains and breeding

Floxed *Tsc1* (*Tsc1* ^*f/f*^) mice were generated in Dr. D. J. Kwiatkowski’s laboratory [23]. In *Tsc1* ^*f/f*^ mice, exons 17 and 18 were flanked with two loxP DNA elements. Exon 17 and 18 deletion eliminated functional Tsc1 protein expression [23]. Inducible lung epithelial cell-specific Cre transgenic mice (SPC-*rtTA*/TetO-*Cre*) were generated in Dr. Jeffrey Whitsett’s laboratory [24]. Heterozygous *Tsc1* knock-down mice SPC-*rtTA*/TetO-*Cre*/*TSC1* ^*f/+*^ (STT) were generated by mating with SPC-*rtTA*, TetO-*Cre* and *Tsc1* ^*f/f*^ mice. ROSA26R transgenic mice bear a lox-STOP-lox-cassette inserted between a promoter and the *LacZ* gene [25]. SPC-*rtTA*/TetO-*Cre*/ROSA26R (STR) mice were generated by crossing with SPC-*rtTA*, TetO-*Cre* and ROSA26R mice. C57BL/6J mice were purchased from Vital River Lab Animal Technology (Beijing, China). The mice were maintained in a specific pathogen free mouse facility at Peking Union Medical College. All animal experiments were approved by the Animal Ethics Committee of Peking Union Medical College according to international and institutional guidelines for animal care. All surgery was performed under sodium pentobarbital anesthesia, and all efforts were made to minimize suffering.

### PCR analysis

All transgenic mice were genotyped by PCR with genomic DNA from mice tails. Primers used for amplifying SPC-*rtTA* transgene were as follows: 5’- GAC ACA TAT AAG ACC CTG GTC A -3’ (forward) and 5’- AAA ATC TTG CCA GCT TTC CCC -3’ (reverse). The expected PCR product was 350 bp. Primers used for amplifying the TetO-*Cre* transgene were as follows: 5’- TGC CAC GAC CAA GTG ACA GCA ATG -3’ (forward) and 5’- AGA GAC GGA AAT CCA TCG CTC G -3’ (reverse). The expected PCR product was 360 bp. Primers used for amplifying ROSA26R were as follows: 5’-GACACCAGACCAACTGGTAATGGTAGCGAC-3’ (forward) and 5’-GCATTGAGCTGGGTAATAAGCGTTGGCAAT-3’ (reverse). The expected PCR product was 750 bp. The *Tsc1* ^*f/f*^ conditional allele was tested using PCR as previously described[23].

For detection of *Tsc1* knock-down, genomic DNA was extracted from lung tissues from STT mice treated with doxycycline. The *Tsc1* knock-down was detected using PCR. The primers for *Tsc1* deletion were as follows: F4536, 5’-AGGAGGCCTCTTCTGCTACC-3’ and R6548, 5’-TGGGTCCTGACCTATCTCCTA-3’ [23]. The expected PCR product size was 370 bp with excision of exons 17 and 18 in the *Tsc1* gene [23].

### Doxycycline administration

In STT mice, Cre-mediated recombination occurred when the inducing agent doxycycline was present. To validate Cre recombinase activity, 4-week-old STT mice and STR mice were administered drinking water containing doxycycline (1 mg/ml) (Sigma) for 10 days. In the bleomycin-mediated lung injury and fibrosis model, 6- to 8-week-old STT and control mice were treated with drinking water containing doxycycline (1 mg/ml) from the day of bleomycin injection and continuously for 21 days until the mice were sacrificed. The drinking water was renewed at 3-day intervals. There were no obvious morphological abnormalities in the lung tissue from doxycycline-treated STT for 4 weeks (data not shown).

### Detection of β-galactosidase activity

For β-galactosidase activity analysis, lung tissues from STR mice were fixed in 0.2% glutaraldehyde and 2% formaldehyde in PBS for overnight at 4°C. After washing three times with PBS at room temperature with gentle shaking, tissue sections were stained overnight in freshly prepared X-gal staining solution containing 2 mM MgCl_2_, 2.5 mM K_3_Fe(CN)_6_, 2.5 mM K_4_Fe(CN)_6_ and 1 mg/ml X-gal. X-gal stock-solution was prepared in dimethylformamide at 40 mg/ml. Stained tissues were then paraffin embedded, and sections were counterstained with nuclear fast red.

### Bleomycin administration and histopathological evaluation of pulmonary fibrosis

Mice (6–8 weeks old) were anesthetized with sodium pentobarbital and administered intra-tracheal (2–3 mg/kg) injections of bleomycin (Nippon Kayaku, Japan) or sterile saline. Mice were sacrificed for analysis by day 21 after bleomycin exposure. The lungs were carefully removed and fixed in 10% formalin overnight, embedded in paraffin, then sectioned at 5μm thickness. The sections were stained with Hematoxylin & Eosin staining or with Masson trichrome staining. Histopathological Ashcroft scoring of pulmonary fibrosis was performed as previously described [26]. The severity of fibrotic changes in each lung section was assessed as a mean score of severity. At least ten high-power fields within each lung section were evaluated. The pathological analysis was performed for each mouse in a blind manner by experienced pathologist.

### Immunohistochemistry

Lung tissue sections (3 μm) were deparaffinized and rehydrated in xylene, heated in 10 mM citrate buffer, and treated with 3% H_2_O_2_ for 10 minutes. After blocking with 5% normal goat serum, these tissue sections were incubated with primary antibodies at 4°C overnight followed by incubation with secondary antibodies for 1 hour at room temperature. The bound secondary antibodies were detected with the Vectastain ABC kit (Vector, USA). Images were taken with a Nikon Eclipse 80i microscope with a digital camera. The primary antibodies used included α-SMA (Abcam) and p-S6 (CST).

### Western blot analysis

Total proteins were extracted from mouse lung tissues or cells. Equal amounts of protein lysates were resolved in a 12% SDS-PAGE gel and blotted onto PVDF membranes (Pierce, USA). The membranes were then incubated with primary antibodies for 1 hour after blocking with 5% nonfat milk in 20 mM Tris (pH 7.5), 0.5 M NaCl, and 0.01% Tween 20 for 1 hour. The blots were then incubated with HRP-linked anti-IgG conjugates for 1 hour at room temperature. Proteins were visualized by enhanced chemiluminescence (Thermo Fisher Scientific Inc.). Primary antibodies used were as follows: α-SMA, p-S6, S6, TSC1, P62, LC3B, GAPDH, and β-actin.

### RNA extraction and RT-PCR

Total RNA was extracted from lungs using TRIzol (Invitrogen). cDNA was synthesized from 2 μg total RNA using the Superscript First-Strand Synthesis System kit (Invitrogen). Quantification of selected genes by semi-quantitative PCR (qPCR) was performed using a LightCycler (Bio-rad). Primer sequences were as follows: Tsc1: TAggTgACAAgCgATAgACT (forward) and gCTgggCACACTCACTTAgT (reverse) and GAPDH: AgggCATCTTgggCTACACT (forward) and ggTCCAgggTTTCTTACTCC (reverse).

### Electron microscopy (EM) analysis

At the 21st day after intra-tracheal bleomycin or saline injection, mice were sacrificed, and lungs were harvested. Lung samples were then fixed with 2.5% glutaraldehyde. Ultra-thin sections were stained with uranyl acetate and lead citrate and analyzed by transmission EM. The total area of autophagosomes and cells was calculated with Adobe Photoshop CS3 Extended software, and the percentage of the cells occupied by autophagosomes was calculated as previously described [27].

### Statistics

For RT-PCR and Western blot analysis, each experiment was repeated with samples obtained from at least 2 different lung or cell preparations. All data are shown as the means ± SEM. Measurements at single time points were analyzed by ANOVA, and if they demonstrated significance, the measurements were further analyzed by a two-tailed t test. Survival data were analyzed by Kaplan-Meier survival analysis. All statistical tests were conducted using GraphPad Prism 5.0 (GraphPadSoftware, San Diego, CA, USA). Findings were considered statistically significant for p< 0.05.

## Results

### mTOR overactivation in fibroblast foci of IPF patients

**Fig 1:**
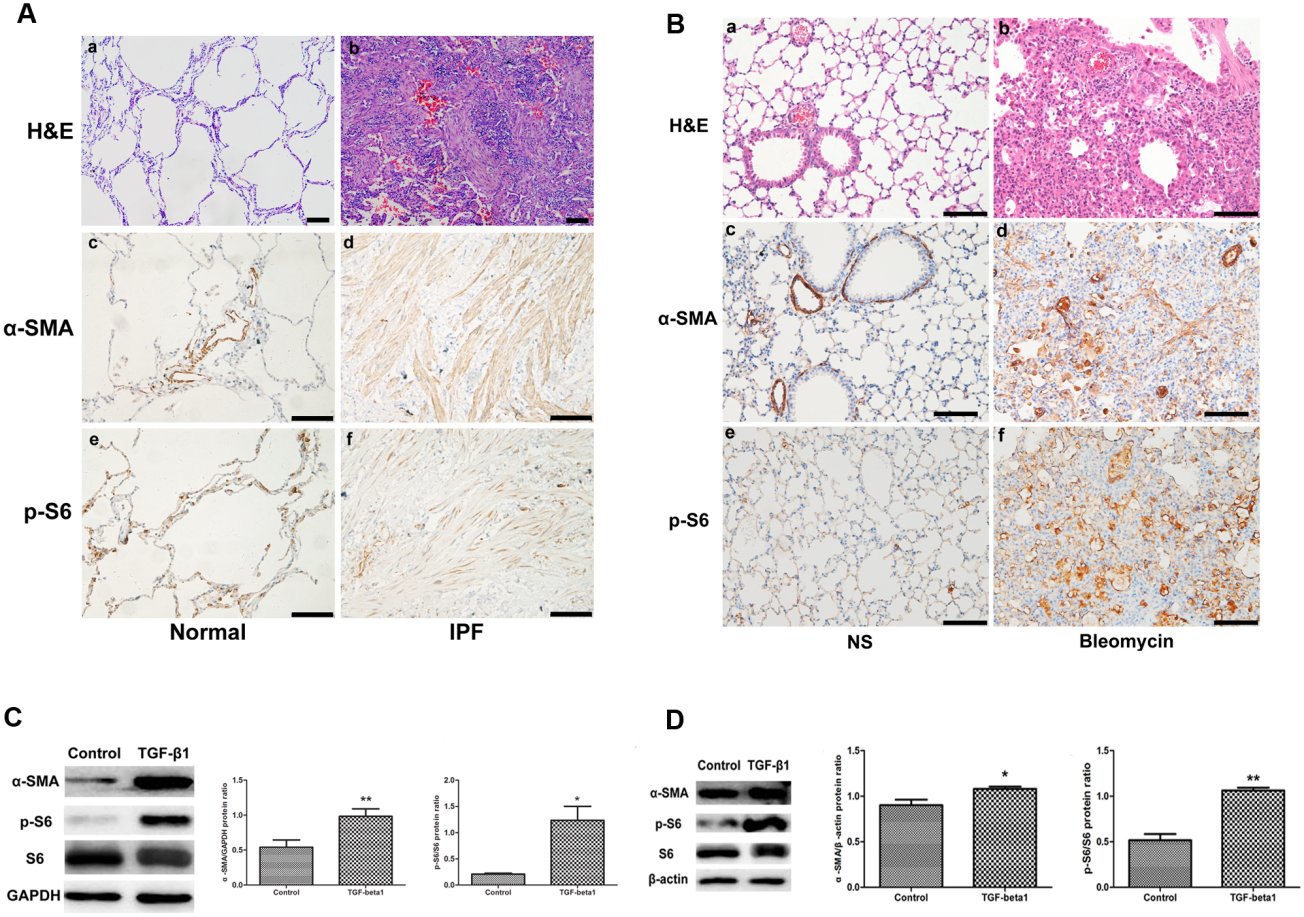
mTOR was activated during the process of pulmonary fibrosis in vivo and vitro. A) mTOR activation in fibroblast foci of lung tissue in IPF patients. a,b, H&E staining with normal control and IPF lung tissues; Immunohistochemical staining performed with α-SMA (c,d) and p-S6 (e,f) antibodies showed an increase in α-SMA and p-S6 in IPF lung tissues (d, f) compared with the control (c, e). Scale bar = 100 μm. B) mTOR activation in the lung tissues of C57BL/6J mice after bleomycin intra-tracheal injection. a,b, H&E staining with lungs of saline and bleomycin-treated mice; Immunohistochemical staining was performed with α-SMA (c,d) and p-S6 (e,f) antibodies in saline- and bleomycin-treated mouse lung tissues. NS, normal saline. Scale bar = 100 μm. C) mTOR signaling pathway was activated in primary lung fibroblasts isolated from normal controls treated with TGF-β1(5 ng/ml) for 48 h. Western blot analysis of α-SMA and p-S6 in control and TGF-β1-treated primary lung fibroblasts (a). Densitometric quantification of the Western blot in (a) is shown in (b) with α-SMA normalized against GAPDH and (c) with p-S6 normalized against S6. **, P<0.01; *, P<0.05. n = 3. D) mTOR signaling pathway was activated in MRC5 cells (a human fetal lung fibroblast cell line) treated with TGF-β1 (5 ng/ml) for 48 h. Western blot analysis of α-SMA and p-S6 in control and TGF-β1-treated MRC5 cells (a). Densitometric quantification of the Western blot in (a) is shown in (b) with α-SMA normalized against β-actin and (c) with p-S6 normalized against S6. **, p<0.01; *, p<0.05. n = 3.

Fibroblast foci are thought to be hallmarks of IPF[28]. To study the mTOR activation in fibroblast foci in IPF lungs, we performed immunohistochemical staining of lungs from IPF patients and healthy controls. α-SMA was mildly expressed in vascular and airway muscle cells in healthy human tissue (Fig 1A-c). In contrast, α-SMA expression was strongly detected in IPF lung tissue (Fig 1A-d), indicating that a large amount of myofibroblasts were transdifferentiated during the IPF process. p-S6, an mTOR downstream effector protein, was observed to have weak expression in healthy lung tissue (Fig 1A-e). However, the p-S6 protein was strongly expressed in myofibroblasts from IPF lung tissue (Fig 1A-f). We concluded that the mTOR signaling pathway was overactivated in fibroblast foci in IPF lung tissue.

### mTOR overactivation is present in the lungs of bleomycin-mediated pulmonary fibrosis

To further verify mTOR activation during the fibrosis process, we performed immunohistochemical staining with α-SMA and p-S6 antibodies in lungs from a bleomycin-mediated pulmonary fibrosis animal model by day 21 after bleomycin intra-tracheal injection in wild-type C57BL/6J mice. α-SMA expression was strongly increased in lungs in bleomycin-treated mice (Fig 1B-d). In contrast, α-SMA proteins were mildly expressed in vascular and airway muscle cells in control mice (Fig 1B-c). p-S6 expression was also more significantly increased in the lungs of bleomycin-treated mice (Fig 1B-f) compared with control mice (Fig 1B-e), indicating that mTOR signaling was also overactivated in the bleomycin-mediated lung injury and fibrosis model.

### mTOR activation is stimulated by recombinant TGF-β1 in isolated primary lung fibroblast and MRC5 cells during fibroblast-myofibroblast transdifferentiation

To explore mTOR activation during the fibrosis process in vitro, we detected α-SMA and p-S6 protein expression during the process of fibroblast-myofibroblast transdifferentiation stimulated by recombinant TGF- β1 in primary lung fibroblast (PLF) and MRC5 cells by Western blot. The PLF cells were isolated from healthy human lungs. After 24 hour stimulation by recombinant TGF-β1 (5 ng/ml) of PLF or MRC5 cells, both α-SMA and p-S6 protein expression was elevated (Fig 1C and 1D), indicating that mTOR signaling is overactivated during the fibrosis process in vitro.

### Conditional Tsc1 knock-down in AECs in SPC-rtTA/TetO-Cre/Tsc1^+/f^ mice

**Fig 2:**
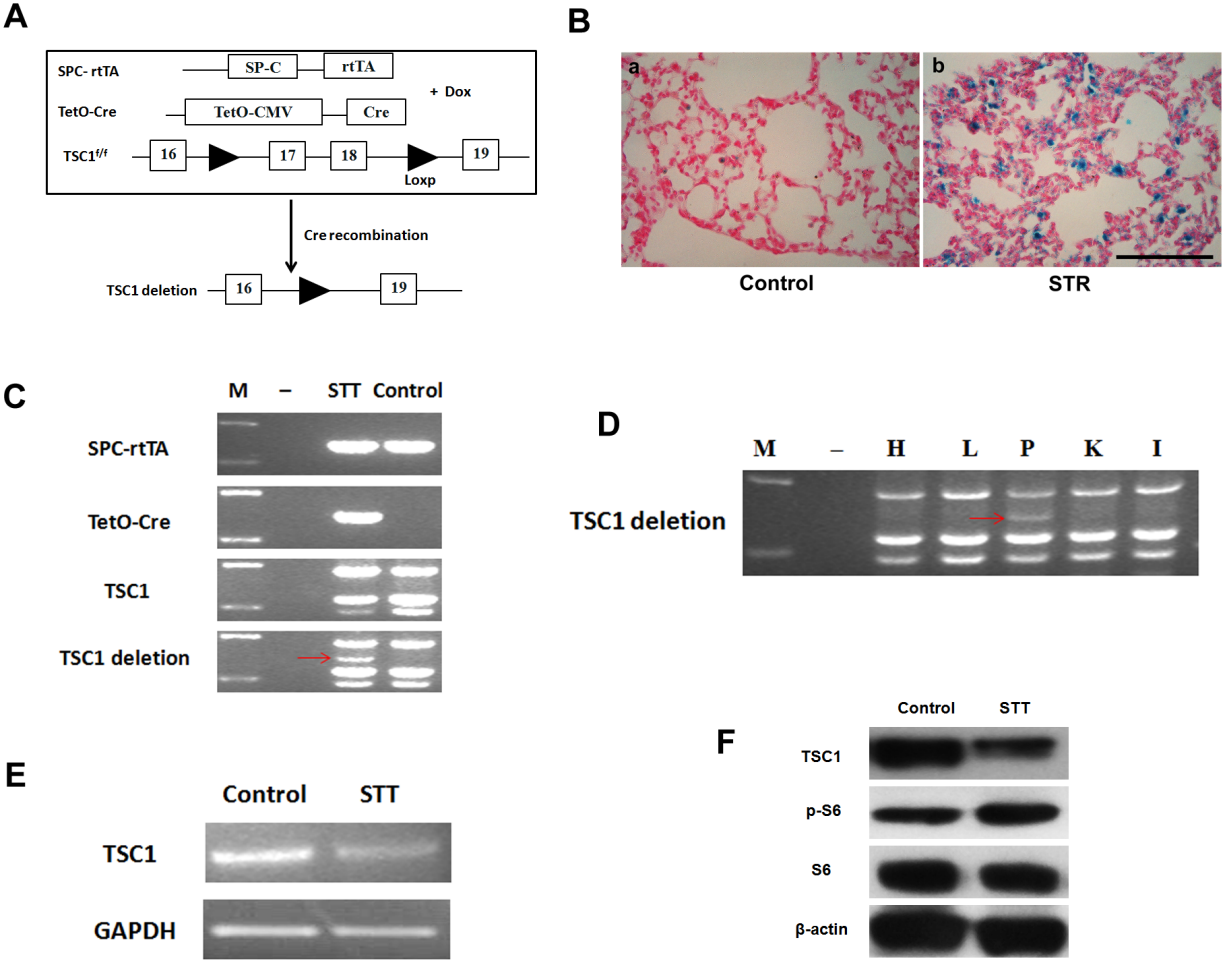
Generation of inducible *Tsc1* knock-down in lung alveolar epithelial cells (SPC-*rtTA*/TetO-*Cre*/*Tsc1* ^*f/+*^ (STT) mice). A) Exons 17 and 18 of the *Tsc1* gene were deleted in alveolar epithelial cells of 4-week-old STT mice by administering doxycycline (Dox). B) β-galactosidase activity was detected in lung alveolar epithelial cells from SPC-*rtTA*/TetO-*Cre*/ROSA26R (STR) transgenic mice after doxycycline treatment (b), while it could not be detected in control mice (a). Scale bar = 100 μm. C) DNA from lung tissues of STT transgenic mice treated with doxycycline were examined by PCR to detect *Tsc1* knock-down, indicating Cre recombinase activity. *Tsc1* deletion band (370 bp, red arrow) was detected in STT mice but not in control mice. M, DNA marker;-, water control. D) The *Tsc1* deletion band (red arrow) was detected in the lungs but not in other organs of a STT transgenic mouse receiving doxycycline treatment. M, DNA marker;-, water control; H, heart; L, liver; P, pulmonary; K, kidney; I, intestine. E) *Tsc1* gene knock-down was confirmed by RT-PCR for *Tsc1* mRNA in lungs from a STT transgenic mouse after doxycycline treatment, and GAPDH was used as the control. F) TSC1 and p-S6 proteins were detected by Western blot in lungs of doxycycline-treated STT transgenic mice. TSC1 was decreased, and p-S6 was elevated, indicating TSC1 knock-down in the lungs of STT mice. S6 and β-actin were used as controls.

To investigate the role of mTOR signaling in AECs during the process of pulmonary fibrosis, we obtained transgenic SPC-*rtTA*/TetO-*Cre*/*TSC1* ^*+/f*^ (STT) mice by crossing three types of transgenic mice, SPC-*rtTA*, TetO-*Cre* and *TSC1* ^*f/f*^, with each other. After treatment with drinking water containing doxycycline (a tetracycline analogue), *Tsc1* knock-down occurred in AECs from STT mice exhibiting deletion of exons 17 and 18 in the *Tsc1* gene through *Cre* recombination (Fig 2A). The *Tsc1* gene encodes hamartin protein (also known as tuberous sclerosis complex 1, TSC1), which forms a complex with TSC2 and regulates the mTORC1 signaling pathway. *Tsc1* deletion can result in activation of the mTOR signaling pathway [5, 23]. To validate the *Cre* recombination in the SPC-*rtTA/*TetO-*Cre* transgenic mouse model, we generated another transgenic mouse line, SPC-*rtTA*/TetO-*Cre*/ROSA26R (STR), by crossing SPC-*rtTA*/TetO-*Cre* transgenic mice with ROSA26R mice. X-gal staining was performed to examine β-galactosidase activity in the lungs of STR transgenic mice after doxycycline treatment. β-galactosidase positive cells were found in the lungs of STR mice but not in control mice (Fig 2B), which confirmed Cre recombination activity in the SPC-rtTA/TetO-Cre model. We also tested doxycycline-induced Cre recombinase activity in STT mice. After treatment with doxycycline, STT mice were sacrificed and lung tissues were harvested. A *Tsc1* deletion band was detected by PCR with genomic DNA from STT mouse lung tissues. We found that the *Tsc1* deletion band was detectable only in STT but not in control mice (Fig 2C), suggesting that Cre recombination occurred in lungs of doxycycline-treated STT mice. Furthermore, we discovered that the *Tsc1* deletion band exists only in the lung but not in the heart, liver, kidney or intestine (Fig 2D), indicating that the SPC promoter has lung tissue-specific activity in the SPC-*rtTA*/TetO-*Cre*/*Tsc1* ^*+/f*^ transgenic mouse. To investigate the TSC1 expression in STT mice, we detected *Tsc1* mRNA and protein expressions in lungs from doxycycline-treated STT mice. We observed that the *Tsc1* mRNA levels were decreased in STT lungs (Fig 2E). TSC1 protein expression decreased, and p-S6 protein expression increased (Fig 2F) in lungs from doxycycline-treated STT mice, indicating that, as a result of *Tsc1* knock-down in lung epithelial cells in STT mice, the mTOR signaling pathway was overactivated.

### Conditional Tsc1 knock-down in AECs exacerbated bleomycin-mediated pulmonary fibrosis and death

**Fig 3:**
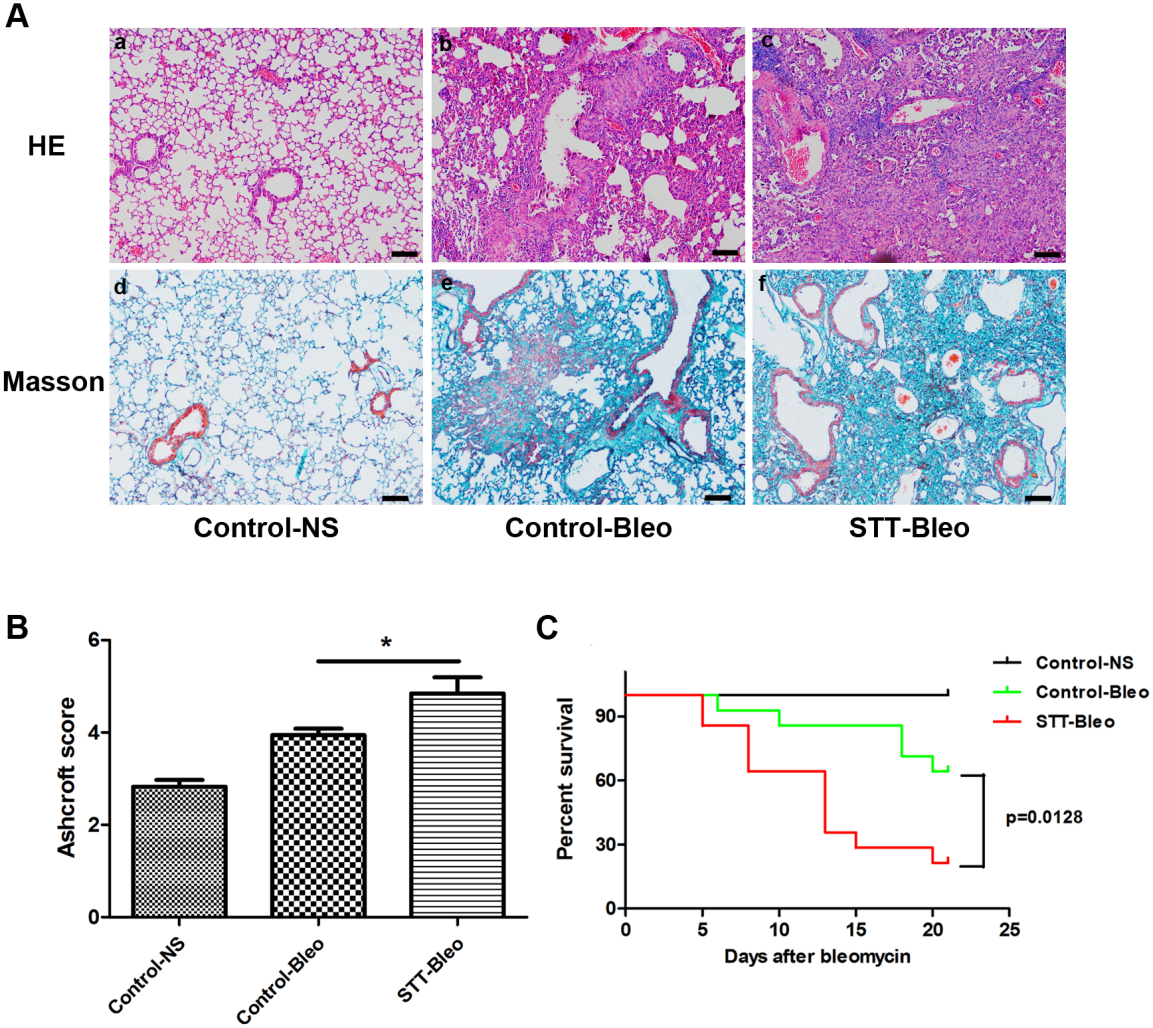
Conditional *Tsc1* knock-down in lung alveolar epithelial cells from doxycyline-treated STT mice exacerbated bleomycin-mediated lung injury. A) Histological analysis of lungs in the mice treated with bleomycin at day 21. H&E staining (a-c) and Masson’s trichrome staining (d-f) were performed. STT mice had more severe lung injury (c, f) than control mice (b, e). Scale bar = 100 μm. NS, saline; Bleo, bleomycin. B) Semi-quantitative assessment was performed on day 21 using Ashcroft scoring method, a significantly higher score was observed in STT mice treated with Bleo than control mice treated with Bleo. Results were expressed as mean±SEM, n = 6 mice per group, * p<0.05. C) STT mice had a higher mortality rate than control mice after a single intra-tracheal injection of bleomycin for 21 days.

Epithelial-specific *Tsc1* knock-down presented a unique opportunity to study the role of mTOR signaling during the fibrosis process in an animal model. Bleomycin was administered at a single dose of 2~2.5 mg/kg by intra-tracheal injection to a group of 8-week-old STT and control mice. We examined the histology of mouse lungs at the 21st day after bleomycin administration through H&E and Masson staining. The STT mice had more severe lung injury and fibrosis than control mice (Fig 3A). STT mice had higher Achcroft scores than control mice (Fig 3B). We found that in the STT mice, intra-tracheal administration of bleomycin caused more death than that for the control mice (Fig 3C). Survival between the two groups was significantly different (p = 0.0128).

### Rapamycin attenuated bleomycin-mediated mouse death and lung injury

**Fig 4:**
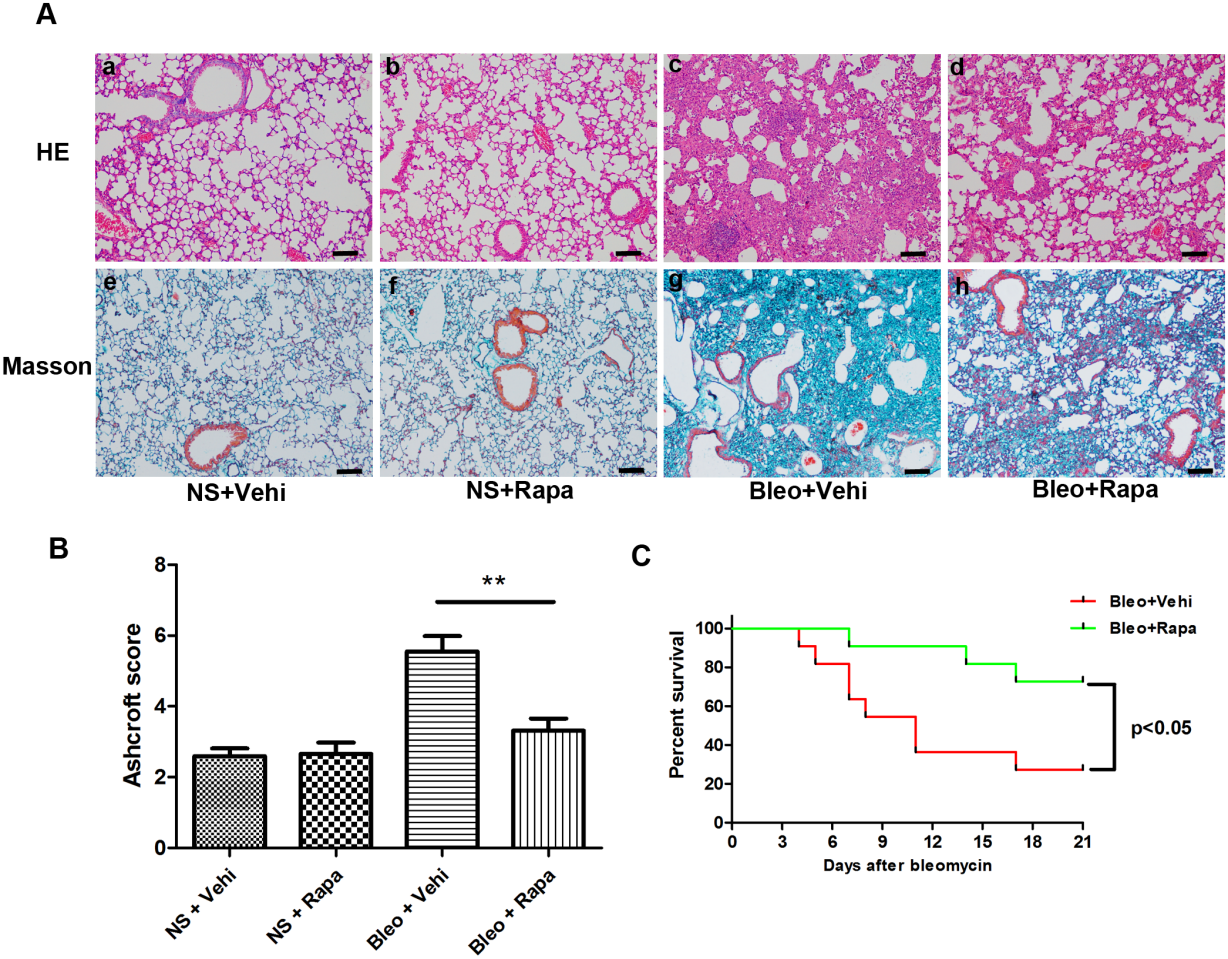
Bleomycin-mediated lung injury in wild-type C57BL/6J mice was attenuated by rapamycin (treatment initiated at 5 days before bleomycin injection). A) H&E staining (a-d) and Masson’s trichrome staining (e-h) of mouse lungs were performed after bleomycin injection at day 21. Lung injury was milder in rapamycin-treated mice (d, h) compared with vehicle-treated mice (c, g). Scale bar = 100 μm. NS, saline; Bleo, bleomycin; Vehi, vehicle; Rapa, rapamycin. B) Semi-quantitative assessment was performed on day 21 using Ashcroft scoring method, a significantly higher score was observed in the mice treated with Bleo (no rapamycin) than those treated with Bleo+Rapa. Results were expressed as mean±SEM, n = 6 mice per group, ** p<0.01. C) Bleomycin-mediated mouse mortality was decreased after rapamycin treatment.

Thus far, we demonstrated that mTOR overactivation in AECs is involved in the pathogenesis of lung fibrosis. We next asked whether mTOR inhibition could decrease the lung injury and fibrosis induced by bleomycin. Rapamycin, an mTOR inhibitor, was reported to be effective in fibrotic diseases, including pulmonary fibrosis [2, 3, 19, 29, 30]. Wild-type C57BL/6J mice were administered a single dose of bleomycin (2–3 mg/kg) by intra-tracheal injection. Treatment with rapamycin (2 mg/kg) or vehicle by intra-peritoneal injection was started 5 days before bleomycin exposure and with subsequent daily injections until the mice were sacrificed. In rapamycin-treated mice, there was less severe lung histology injury and fibrosis than that observed with vehicle control mice (Fig 4A). As shown in Fig 4B, there were significantly higher histopathologic scores in the mice treated with bleomycin (no rapamycin) than scores in the mice treated with rapamycin and bleomycin (Fig 4B, p<0.01). The survival rates between two groups were significantly different (Fig 4C, p<0.05). Rapamycin attenuated bleomycin-mediated lung injury and death in mice, further supporting the idea that mTOR overactivation is involved in the process of pulmonary fibrosis.

### Rapamycin protects mice from bleomycin-mediated lung injury by upregulating autophagy, which could be reversed by chloroquine

**Fig 5:**
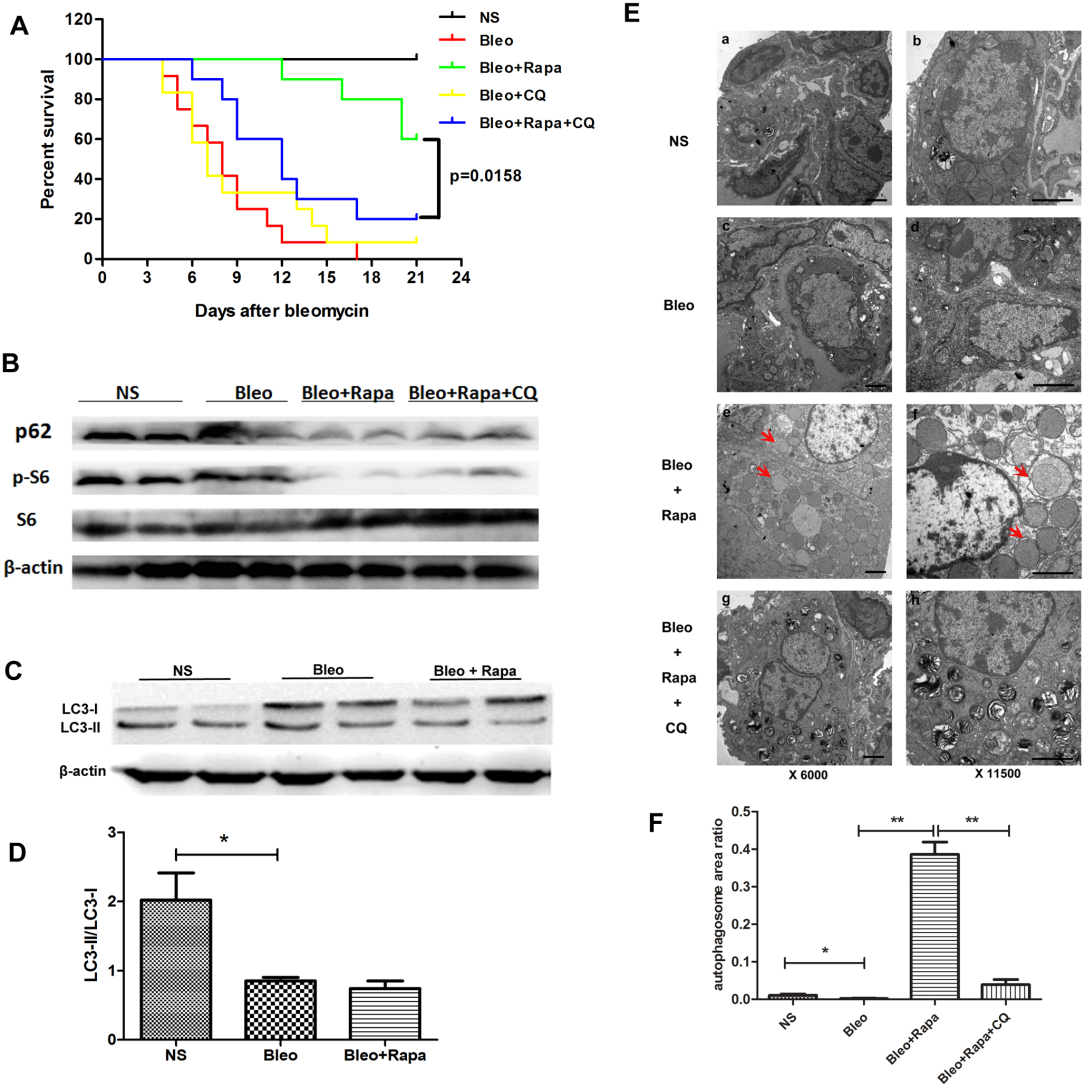
Rapamycin-induced autophagyin the bleomycin-mediated lung injury and fibrosis model. A) Rapamycin decreased the death caused by bleomycin. Chloroquine, an autophagy inhibitor, reversed the benefit of rapamycin in the bleomycin-mediated lung injury model (Bleo+Rapa+CQ vs Bleo+Rapa, p = 0.0158). B) Western blot analysis of p62 and p-S6 were performed in the bleomycin-mediated lung injury and fibrosis model. p62, a protein inversely correlated with autophagy activity, was decreased in lungs of mice treated with rapamycin alone. p62 expression was higher with combined rapamycin and chloroquine treatment than with rapamycin alone. S6 and β-actin were used as controls. C) Western blot ananlysis of LC3 I and LC3 II were performed in the bleomycin-mediated lung injury and fibrosis mice model. D) Relative density of LC3 II/LC3 I of bands in Fig 5C. Autophagy was significantly decreased in bleomycin-mediated lung injury and fibrosis model (*bleomycin vs normal saline, p < 0.05). E) Electron microscope images of lung tissues show autophagosomes in the bleomycin-mediated lung injury model. Arrows indicate autophagosomes. Rapamycin treatment alone induced an increased number of autophagosomes. Left panel, original magnification: 6,000X and right panel, original magnification: 11,500X. F) Statistical results for the autophagosomes in Fig 5E. The statistical results indicate the percent area of autophagosomes in a cell.

Autophagy is a major mechanism for maintaining cellular homeostasis via autophagic cell death [31]. Relationships between autophagy and the mTOR signaling pathway have been previously reported [32, 33]. Autophagy might play important role in the pathogenesis of lung diseases, including pulmonary fibrosis [34, 35]. Chloroquine, an anti-malarial drug, was thought to be effective in the treatment of H5N1-mediated lung injury as an autophagy inhibitor [27]. We found that chloroquine could not rescue bleomycin-mediated mouse death (Fig 5A). Combined rapamycin and chloroquine treatment demonstrated more severe mortality than rapamycin alone (p = 0.0158, Fig 5A). We also detected p62, a protein inversely correlated with autophagy activity, by Western blot in lung tissue. p62 expression was increased in the lungs of bleomycin-treated mice (Fig 5B). p62 expression was decreased in rapamycin-treated mice, and it could be elevated by chloroquine treatment (Fig 5B). LC3 II/LC3 I ratio, an indicator of autophagy activity, was significantly decreased in bleomycin-treated lung (Fig 5C and 5D). Autophagosomes were examined further by EM in lungs from the bleomycin-mediated lung injury and fibrosis model. Rapamycin induced autophagosome production in mouse lungs and could be decreased by chloroquine (Fig 5E and 5F).

## Discussion

In this report, we demonstrated that aberrant mTOR signaling pathway activation in AECs and insufficient autophagy involve in the pathogenesis of lung injury and fibrosis. We studied whether mTOR was involved in the process of pulmonary fibrosis in human IPF lung tissues, an animal model of bleomycin-mediated pulmonary fibrosis in vivo, and during trans-differentiation from fibroblasts to myofibroblasts in vitro. We specifically demonstrated the role of abnormal mTOR activity in AECs using a conditional *Tsc1* knock-down mouse model. Then, we explored whether mTOR inhibition could rescue bleomycin-mediated lung injury and fibrosis. Furthermore, we investigated the autophagy signaling pathway in a bleomycin-mediated lung fibrosis model, establishing a functional link between mTOR signaling and autophagy in lung injury and fibrosis.

Growing evidences support the hypothesis that mTOR overactivation is involved in the pathogenesis of fibrotic diseases, including pulmonary fibrosis [13, 17, 19]. Repetitive injury and repair of AECs are regarded as critical mechanisms of pulmonary fibrosis. We specifically focused on the role of mTOR overactivation in AECs using an induced conditional knock-down mouse model. Our data further demonstrated that the mTOR inhibitor rapamycin attenuated bleomycin-mediated lung injury and mortality (Fig 4), which is consistent with a previous study exhibiting that mTOR inhibitors are effective for treating pulmonary fibrosis in animal models[18–20, 36]. After obtaining strong evidence supporting our hypothesis that mTOR overactivation was involved in the process of pulmonary fibrosis, we further demonstrated that mTOR activation-related autophagy insufficiency could be a major mechanism.

The potential application of mTOR inhibitors for pulmonary fibrosis was discouraged by a clinical study that showed everolimus, another mTOR inhibitor other than rapamycin, was associated with more rapid disease progression in patients with surgical lung biopsy-confirmed IPF[37]. Efficacy of mTOR inhibitors in treating IPF appears to be controversial. In this study, we used a rapamycin pre-treatment strategy (initiated at 5 days before bleomycin injection) in a bleomycin-mediated lung fibrosis model. In addition, we also used a rapamycin late-treatment strategy (initiated at 8 days after bleomycin injection), which mimics clinical practice. However, the rapamycin late-treatment strategy exhibited no benefit for lung injury and survival (data not shown). Another animal study showed that early rapamycin treatment (one day before bleomycin injection) caused a significant decrease in fibrosis score, while late rapamycin treatment (nine days after bleomycin injection) did not [20]. Even though, mTOR inhibitors are still potential candidates for the treatment of IPF. Overall, the information of appropriate dosage and timing of sirolimus for animal models of pulmonary fibrosis and patients are lacking. Sirolimus would be more effective in prevention of disease progression, but less effective for established fibrosis. Clinically, a case report described the successful treatment of a severe IPF patient with rapamycin [21]. Clarification of how to effectively use mTOR inhibitors in patients is required, i.e., dosage, timing and duration, route of administration (inhalation or by oral), biomarkers, and effectiveness in different clinical and genetic backgrounds. A double-blind placebo-controlled study of sirolimus (rapamycin) for IPF is ongoing (ClinicalTrials.gov Identifier number: gov NCT 01462006).

Alveolar epithelial injury and aberrant repair are regarded as key mechanisms in the pathogenesis of pulmonary fibrosis [22]. Abnormal proliferation, apoptosis, injury and repair in AECs are thought to be mechanisms for IPF [38, 39]. AECs have been believed to be an origin for myofibroblasts during the lung fibrosis process [40], but it has been reported that this is unlikely to be true [41]. Nevertheless, epithelial-dependent myofibroblast activation should be an important mechanism in the pathogenesis of pulmonary fibrosis. Using a conditional knock-down mouse model, we had the unique opportunity to explore whether aberrant mTOR signaling pathway in AECs plays a critical role in lung fibrosis. In STT mice, *Tsc1* knock-down occurred in AECs after doxycycline treatment, resulting in mTOR overactivation. We found that mTOR overactivation in AECs aggravated bleomycin-mediated lung injury and fibrosis (Fig 3). These results demonstrated that abnormal mTOR activity in AECs plays an important role in the pathogenesis of pulmonary fibrosis. Using a similar approach, *Pten* deletion in AECs demonstrated critical importance in lung injury and fibrosis [42]. The mechanisms of interaction between AECs and fibroblasts require further investigation. Anyway, the Pten/PI3K/Akt/mTOR pathway provides multiple potential novel targets for the treatment of IPF.

Autophagy, another core signaling pathway maintaining cellular hemostasis, has been recently reported in the pathogenesis of pulmonary diseases [36, 43]. Macroautophagy involves the formation of a double membrane around the organelle known as autophagosomes. Decreased autophagy was found in lung tissues from IPF patients [34]. Another study showed p62 expression in human IPF lung tissue through immunohistochemical evaluation, revealing decreased autophagic activity in IPF [35]. Decreased expression of the beclin 1 autophagy protein was found in IPF fibroblasts [44]. The data is quite consistent in supporting the idea of potential significance of insufficient autophagy in IPF. Rapamycin, as a specific mTOR inhibitor, was shown to induce autophagy activity in previous reports [32, 33, 45]. Thus, we deduced that the beneficial effects of rapamycin in pulmonary fibrosis might be through autophagy induction. In this study, we demonstrated increased p62, decreased LC3 II/LC3 I ratio, and decreased autophagosomes in lungs from a bleomycin-mediated lung fibrosis model (Fig 5). Pre-treatment with rapamycin attenuated the mortality of mice accompanying with autophagosome production and p62 reduction (Fig 5). Furthermore, the beneficial effects of rapamycin could be reversed by chloroquine, an autophagy inhibitor (Fig 5), supporting the idea that the beneficial effects of rapamycin for pulmonary fibrosis might be through the mechanism of autophagy induction. Enhancement of autophagy depending or not depending on mTOR inhibition worth to be further investigated in pulmonary fibrosis.

## Conclusion

mTOR overactivation is involved in pathogenesis of pulmonary fibrosis via multiple cell types including AECs and fibroblasts. mTOR overactivation in AECs and dysfunctional autophagy involve in the pathogenesis of pulmonary fibrosis. Inhibition of mTOR overactivation and the induction of autophagy are potential treatment for IPF.
